# Effects of physical activity on endothelial progenitor cells (EPCs)

**DOI:** 10.3389/fphys.2013.00414

**Published:** 2014-02-03

**Authors:** Chiara De Biase, Roberta De Rosa, Rossella Luciano, Stefania De Luca, Ernesto Capuano, Bruno Trimarco, Gennaro Galasso

**Affiliations:** Department of Advanced Biomedical Sciences, “Federico II” University of NaplesNaples, Italy

**Keywords:** cardiovascular disease, physical activity, endothelial progenitor cell, coronary artery disease, bone marrow

## Abstract

Physical activity has a therapeutic role in cardiovascular disease (CVD), through its beneficial effects on endothelial function and cardiovascular system. Circulating endothelial progenitor cells (EPCs) are bone marrow (BM) derived cells that represent a novel therapeutic target in CVD patients, because of their ability to home to sites of ischemic injury and repair the damaged vessels. Several studies show that physical activity results in a significant increase in circulating EPCs, and, in particular, there are some evidence of the beneficial exercise-induced effects on EPCs activity in CVD settings, including coronary artery disease (CAD), heart failure (HF), and peripheral artery disease (PAD). The aim of this paper is to review the current evidence about the beneficial effects of physical exercise on endothelial function and EPCs levels and activity in both healthy subjects and patients with CVD.

## Introduction

Physical activity improves endothelial function both in healthy subjects and in patients with cardiovascular disease (CVD) (Volaklis et al., 2013) through several favorable effects on inflammation and regulation of autonomic tone and blood pressure. Endothelial progenitor cells (EPCs) are bone marrow (BM) derived cells that, following endothelial damage, are recruited into systemic circulation and home to sites of ischemic injury (Galasso et al., 2013), where they contribute to vascular repair. It has been reported that EPCs have regenerative and proliferative potential, and act as “software” that promote neovascularization through secretion of pro-angiogenic cytokines (Heil et al., 2004). EPCs number and function are inversely correlated with age and common cardiovascular risk factors like diabetes, smoking, hypertension, and hyperlipidemia (Vasa et al., 2001; Hill et al., 2003). Low levels and impaired activity of circulating EPCs have been shown to be an independent predictor of morbidity and mortality in patients with CVD (Dzau et al., 2005), whereas high levels of EPCs have been associated with longer event-free survival from adverse cardiovascular events (Werner et al., 2005; Cassese et al., 2013). Several studies demonstrated that a regular physical activity has a positive effect on the levels of circulating EPCs, by inducing EPCs mobilization from the BM niche (George et al., 2011) and counteracting EPCs impairment in the presence of cardiovascular risk factors (Volaklis et al., 2013). In this paper, we aim to review the current evidence about the effects of physical activity on endothelial function and EPCs number and activity, in healthy patients and in the setting of CVD.

## Characterization and role of EPCs

In 1997 Asahara and colleagues first isolated and characterized putative EPCs from human peripheral blood, and established their ability to form clusters of round cells on fibronectin-coated dishes (Asahara et al., 1997), and their regenerative potential regarding neoangiogenesis and vascular repair. Afterwards Hill et al. defined putative EPCs as colony forming unit-Hill (CFU-Hill), and showed a significant inverse correlation between circulating CFU-Hill number and Framingham cardiovascular risk score in humans (Hill et al., 2003). Nevertheless, up to date the proper definition of EPCs is still a matter of debate and EPCs characterization is performed according to AHA recommendation (Hirschi et al., 2008; Yoder, 2010), defining a human EPC as a circulating cell that promotes neovascularization at sites of ischemia, hypoxia, injury, or tumor formation (Hirschi et al., 2008). The majority of EPCs reside in the BM, in close association with hematopoietic stem cells (HSCs) and BM stromal cells that provide the microenvironment for hematopoiesis (Luttun et al., 2002), representing only 0.02% of circulating mononuclear cells (MNC) in peripheral blood (Galasso et al., 2006). The mobilization of EPCs from the BM niche is known as “recruitment”. There are many stimuli able to promote EPCs recruitment (Dimmeler et al., 2001; Aicher et al., 2005; Schiekofer et al., 2008; Jung et al., 2009), including pro-angiogenic growth factors like angiopoietin-1, fibroblast growth factor and stromal cell-derived growth factor-1 (SDF-1) (Yamaguchi et al., 2003). Several studies have shown that the vascular endothelial growth factor (VEGF), or rather, the most common isophorm of VEGF, VEGF-165, is the main player in promoting EPCs mobilization and their incorporation into sites of neovascularization. Moreover, VEGF-165 induces EPCs proliferation and modulates the expression of adhesion molecules that promote EPCs recruitment from the BM. Indeed, it has been recently demonstrated that a *de novo* engineered VEGF mimicking peptide, known as QK, acts in the same biological way of VEGF, improving capillary formation both *in vitro* and *in vivo* (Santulli et al., 2009). An important role is also played by endothelial-nitric oxide synthase (e-NOS), expressed by BM stromal cells, that influences EPCs recruitment and migration through modulation of MMP-9 and production of NO (Schmidt and Walter, 1994; Aicher et al., 2003). Furthermore there are many cytokines, chemokines and drugs supporting the EPCs homing to sites of re-endothelialization and the EPCs incorporation into sites of vascular injury (Shi et al., 1998; Baller et al., 1999; Eliceiri and Cheresh, 1999; Peichev et al., 2000; Walter et al., 2002; Cittadini et al., 2009; Strisciuglio et al., 2012). This means that EPCs participate in the maintenance of vascular homeostasis by restoring an intact endothelium and acting as the substrate for new vessel formation promoting neoangiogenesis. Noteworthy, physical activity seems to be a further stimulus to induce EPCs recruitment and homing, improving several mechanisms underlying EPCs mobilization (Leone et al., 2009).

## Mechanisms of physical activity-induced changes on EPCs

**Figure 1:**
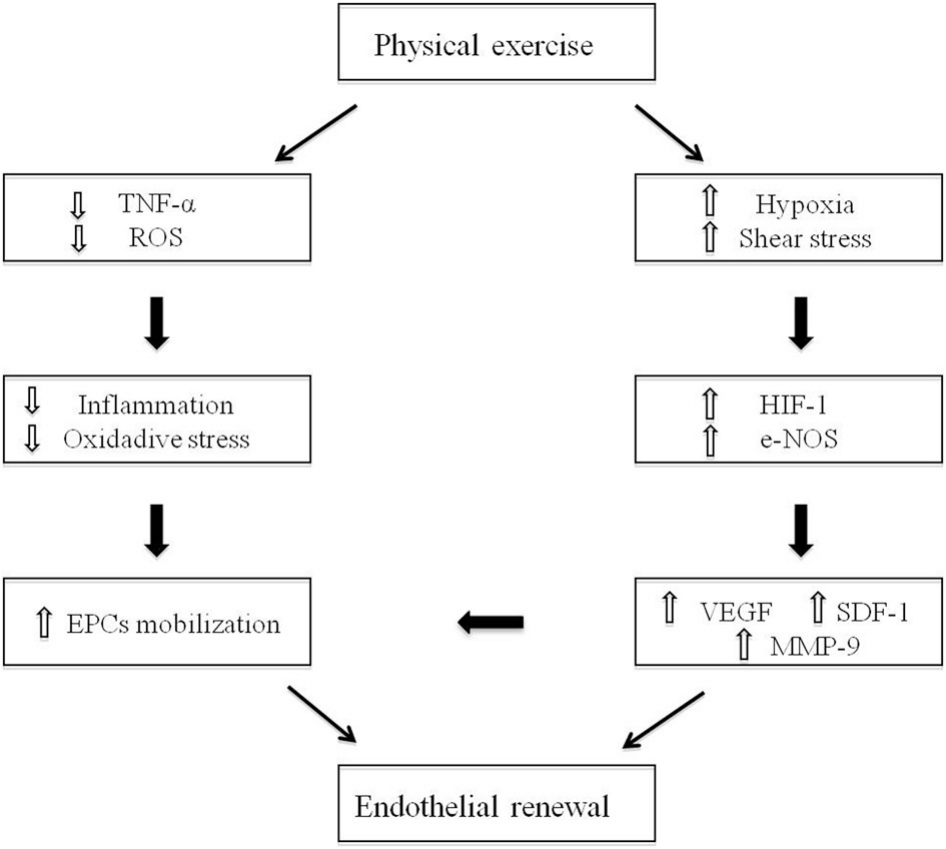
**Effects of physical exercise on endothelial function and EPCs**.

Physical activity is a potent inductor of EPCs mobilization from the BM niche and promotes homing of these cells to sites of ischemia (Leosco et al., 2008; Leone et al., 2009; Ribeiro et al., 2013) (Figure 1). The effects of physical exercise training on endothelial function and EPCs activity have been investigated by several studies, in animal or human models, undergoing both physical active and sedentary lifestyle (Table 1). It has been reported that both acute and chronic exercise lead to the increase of circulating EPCs, thus highlighting the main role of exercise intensity and duration on EPCs mobilization (Laufs et al., 2005; Hoetzer et al., 2007; Van Craenenbroeck et al., 2008; Jenkins et al., 2009; Volaklis et al., 2013). Moreover, the exercise-induced alterations in vascular shear stress, with increase in blood flow and e-NOS activity, act as potent stimulus to EPCs release from the BM. Interestingly, mice lacking the e-NOS gene showed a reduction in circulating EPCs number and function beyond endothelial dysfunction (Huang et al., 1995; Cooke and Dzau, 1997; Kojda et al., 2001; Aicher et al., 2003). Accordingly, some evidence from studies conducted both in trained mice (Laufs et al., 2004) and in human patients undergoing exercise tests (Rehman et al., 2004), showed an increase in e-NOS activity and EPCs levels after exercise training. Furthermore, it has been demonstrated that exercise-induced ischemia increases VEGF levels in serum, mainly through induction of hypoxia-inducible factor 1 (HIF-1) (Forsythe et al., 1996), with consequent EPCs mobilization. These findings are prominent in sedentary old population (Taddei et al., 1995; Gerhard et al., 1996), since the effects of aging on EPCs disability are related to both the senescence of EPCs and the down-regulation of pro-angiogenic factors like HIF-1 and VEGF (Torella et al., 2004; Leosco et al., 2007a). Interestingly, physical exercise can prevent and reverse age-related endothelial dysfunction, representing a valid strategy to stimulate EPCs in old subjects (DeSouza et al., 2000; Smith et al., 2003; Heiss et al., 2005; Hoetzer et al., 2007; Yang et al., 2013). In addition, physical exercise leads to a significant reduction of myelosuppressive and pro-inflammatory cytokines, like C-reactive protein (CRP) (Szmitko et al., 2003) and tumor necrosis factor-α (TNF-α) (Agnoletti et al., 1999), thus exerting also an anti-inflammatory role. It is well known that in pathological condition, such as ischemia, there are high levels of circulating inflammatory cytokines and an increase of radical oxygen species (ROS) production, with consequent NO inactivation (Ross, 1999; Brevetti et al., 2008), EPCs apoptosis (Galasso et al., 2006) and endothelial dysfunction. EPCs contain high levels of ROS-metabolizing enzymes that are essential to maintain their survival during tissue regeneration under conditions of injury (Raes et al., 1987). Noteworthy, in models of glutathione peroxidase type 1 (GPx-1)-deficient mice, EPCs were functionally impaired, with consequent deficiency of ischemia-induced angiogenesis (Galasso et al., 2006). Physical activity can counteract both the lack of NO availability and the vascular oxidative stress, by increasing extracellular superoxide dismutase (SOD) with enhancement of vascular repair and angiogenesis, and by reducing pro-inflammatory cytokines and ROS production with longer EPCs survival (Fukai et al., 2000). Accordingly, human ECs, when conditioned with sera of triathletes practicing a moderate physical activity, showed a better proliferative potential and a longer survival (Conti et al., 2012, 2013). Furthermore, exercise improves endothelial and EPCs function through the activation of the adrenergic vascular system (Barbato et al., 2005; Iaccarino et al., 2005; Ciccarelli et al., 2008; Piscione et al., 2008). Indeed, the exercise-induced adrenergic stimulation results in a significant reduction of cardiac β2-adrenergic-receptors (β2-AR) down-regulation and desensitization in patients with CVD, two mechanisms underlying the impaired endothelial vasodilation and the vascular dysfunction in this clinical setting (Iaccarino et al., 2002; Leosco et al., 2007b; Lymperopoulos et al., 2007; Ciccarelli et al., 2008; Rengo et al., 2011; Sorriento et al., 2011; Lymperopoulos et al., 2012). We recently showed that β2-ARs stimulation could also induce an increase in EPCs number, acting on the EPCs fraction of circulating MNC to promote EPCs differentiation, and ameliorating EPCs-induced neoangiogenesis both *in vitro* and *in vivo* (Galasso et al., 2013). Indeed, it was shown that, in aged animal, myocardium exercise attenuated the age-induced β2-ARs dysfunction by modulating the G-coupled receptor-kynase-2 (GRK-2) levels (Leosco et al., 2003; Santulli et al., 2013). Nevertheless, the strongest evidences of beneficial exercise-induced effects on EPCs function and numbers derive from studies conducted in CVD settings, including coronary artery disease (CAD), heart failure (HF) and peripheral artery disease (PAD).

**Table 1:** **Summary of the studies reporting the exercise induced effects on EPCs, in animal and human models**.

**Study**	**Physical/health status**	**Exercise program**	**Principal findings**
Hoetzer et al. (2007)	Sedentary	40–50 min at 65–85% of HRR_*max*_/aerobic	EPCs migratory activity enhanced +49.6%
Laufs et al. (2005)	Healthy	Three different exercise protocols 30 min at 82%; 30 min at 68%; 10 min at 68% of VO_2*max*_	Significant increases in EPCs only after 30 min protocols (+235% and +263%, respectively)
Van Craenenbroeck et al. (2008)	Healthy	Maximal cardiopulmonary exercise testing	Significant increase in EPCs (+76% in young vs. +69% in old)
Laufs et al. (2004)	Animal models	30 min with 12 m/min/aerobic	EPCs enhanced +267, +289, and +280% after 7, 14, 28 days of training respectively; reduction of EPCs apoptosis and inhibition of neo-intima formation
Laufs et al. (2004)	CAD	15–20 min at 60–80 of VO_2*peak*_/aerobic	EPCs increased by 78%; apoptosis rate reduced by 41%
Rehman et al. (2004)	Chronic disease/sedentary	Symptom-limited exercise stress test until 90% of HR_*peak*_	Significant increase in EPCs (+258%)
Yang et al. (2013)	Healthy young (25 ± 1 years)	Three months of regular exercise training 3 times/week for 30 min	The age related decline in the EPCs number, migratory and proliferative activity enhanced less in endurance-trained men
	Healthy old (61 ± 2 years)		
	Sedentary/endurance trained		
Adams et al. (2004)	Ischemic CAD	Maximal stress testing	Significant increase in EPCs (+164.0%) up to 6 h after exercise only in the ischemic patients
	Non ischemic CAD		
Scalone et al. (2013)	MVA	Exercise stress test	ECFC increased less in MVA patients after exercise
	CAD		
Cesari et al. (2013)	ACS	Five weeks of cardiac rehabilitation program	EPCs enhanced respect to baseline; hs-CRP and NT-ProBNP decreased
Ikeda et al. (2008)	ACS	30–60 min walk daily 4 times/week by 11 days after ACS	EPCs number and exercise capacity enhanced at 3 months from ACS
Sarto et al. (2007)	Heart failure (EF% 30.5%)	55 min at 60% of HRR/aerobic	EPCs enhanced +251%
Van Craenenbroeck et al. (2010)	Chronic Heart failure	Six months exercise training	EPCs enhanced and endothelial function improved
Mezzani et al. (2013)	Heart failure	Light to moderate intensity aerobic exercise training	EPCs enhanced by 9, 20, and 98% respectively at phase I, phase IIT and mean response time
Erbs et al. (2010)	Heart failure (EF 24%)	5–20 min at 50% of VO_2*max*_/aerobic 20–30 min at 60% of VO_2*max*_/aerobic	EPCs enhanced +80% and EPCs migratory activity enhanced +224%
Sandri et al. (2005)	PAD	Aerobic training	Significant increase in EPCs only in PAD patients
	Prior-PAD		
	CAD		
Schlager et al. (2011)	PAD	Two sessions of 50 min walking at speed eliciting claudication symptoms 2 times/week for 6 months	EPCs enhanced; ADMA decreased

EPCs, endothelial progenitor cells; CAD, coronary artery disease; PAD, peripheral artery disease; EF, ejection fraction; HRR, heart rate reserve; MVA, micro-vascular angina; ECFC, endothelial colony-forming cells; ACS, acute coronary syndrome; hs-CRP, high sensivity C-reactive protein; NT-ProBNP, N-Terminal Pro-Brain Natriuretic Peptide; ADMA, asymmetric dimethylarginine.

### Effects of physical activity on EPCs in CAD

In human setting of stable CAD, it was demonstrated that both 28 days of moderate exercise training and 12 weeks of running protocols led to reduced EPCs apoptosis and increased circulating EPCs levels (Laufs et al., 2004; Hoetzer et al., 2007). Moreover, confirming these results, further studies demonstrated that the EPCs concentration in peripheral blood after maximal stress test in CAD patients resulted significantly enhanced and correlated with increase in VEGF and NO release, improving vascular flow-mediated dilatation (FMD) (Adams et al., 2004). To examine in depth the effects of physical exercise on EPCs in CAD prevention, EPCs function was evaluated also in patients with micro-vascular angina (MVA) and CAD, both at rest and 24 h after exercise stress test (EST). Results showed lower EPCs levels in CAD subjects at rest, while there was an increased EPCs count after 24 h of exercise, both in MVA and CAD patients (Scalone et al., 2013). Accordingly, in patients with acute coronary syndrome (ACS) there was an increase in EPCs levels, accompanied with a significant decrease in pro-inflammatory biomarkers, after 1 month of cardiac rehabilitation (CR) program on a cycle-ergometer (Cesari et al., 2013). Moreover, an exercise program of 30–60 min walk per day, starting 11 days after ACS, induced increment of EPCs number in male patients with acute myocardial infarction (AMI) (Ikeda et al., 2008). In conclusion, the aforementioned evidence highlight that physical training exerts beneficial effects on vascular integrity and on endothelial function in condition of either mild or severe coronary atherosclerosis, improving the outcome of patients with CAD (Rengo et al., 2007, 2010).

### Effects of physical activity on EPCs in HF

EPCs have been hypothesized to realize a compensatory increase in patients with mild to moderate HF, in order to reduce vascular damage of impaired heart (Valgimigli et al., 2004; Piscione et al., 2005). Indeed, physical activity induces the activation of the cardiac VEGF pathway, with enhancement of myocardial angiogenesis, significant increase in myocardial perfusion and coronary flow reserve, and improvement in left ventricular contractility. Studies conducted in patients with HF and impaired endogenous endothelial regenerative capacity, evaluated the effects of training and detraining on circulating EPCs levels. After both 8 weeks or 6 months of aerobic physical activity, there was an increased number of EPCs, accompanied with higher VEGF and SDF-1 levels in plasma of trained HF patients, while EPCs number returned to baseline when analyzed after a appropriate period of detraining (Sarto et al., 2007; Van Craenenbroeck et al., 2010, 2011). It has been further demonstrated that NYHA class II patients, randomized to 3 months exercise or to control group, showed different levels of circulating EPCs in response to physical training, even if there was no difference of EPCs number in exercised or control group at baseline (Mezzani et al., 2013). In addition, even in severe HF patients with reduction of left ventricular ejection fraction (LVEF), randomly assigned to 12 weeks of physical training or sedentary lifestyle, exercise induced improvement in EPCs count and migratory capacity, associated with enhancement in neovascularization of skeletal muscle and ejection fraction (Erbs et al., 2010). On the whole, the evidence suggest that exercise training can induce optimization of EPCs blood levels also in patients with severe impairment of LVFE, representing a potential mechanism to improve life quality of this subgroup of patients.

### Effects of physical activity on EPCs in PAD

Additional studies confirmed that aerobic physical exercise exerted beneficial effects on EPCs number and activity also in patients with PAD. Indeed, it was analyzed the mobilization of EPCs, after 4 weeks of daily aerobic exercise, in three randomized controlled studies, including PAD patients, PAD patients after successful revascularization, and stable CAD patients, and the results showed a significant increase in EPCs blood count, in particular in PAD patients subgroup (Sandri et al., 2005). Accordingly, significant enhancement in EPCs levels were found in subjects with PAD assigned to exercise or control group, even if EPCs number decreased within 6 months after training interruption (Schlager et al., 2011). All these results suggest that physical activity improves endothelial function and EPCs number and activity, and outline that acute exercise in healthy as in diseased individuals can increase the primarily EPCs recruitment from BM, even if a sustained physical activity is necessary to preserve these ameliorations (Haram et al., 2008).

## Conclusions

Improvement of EPCs number and pro-angiogenic activity represents an innovative target to counteract negative effect of aging and CVD. Several researches show that physical activity, performed as part of an exercise-training program, results in a significant increase in circulating EPCs levels and function. Accordingly, significant improvement of endothelial function has been demonstrated in patients with CVD who experienced exercise training. Indeed, EPCs mediated neovascularization could represent a useful way to get a therapeutic revascularization of ischemic areas in patients with endothelial dysfunction and high cardiovascular risk (Isner and Asahara, 1999). In particular, EPCs therapy could represent an interesting therapeutic option in the management of patients with stable CAD or previous myocardial infarction (MI), by increasing tissue perfusion in the ischemic area and rescue hibernating myocardium (Piscione et al., 2011). However, further studies are required to investigate the effects of exercise training on EPCs activity in both healthy subjects and patients with cardiovascular risk factors, and to refine the best protocol of exercise-training to up regulate circulating EPCs, clarifying the kinetics of EPCs after the suspension of different exercise sessions (Volaklis et al., 2013). On the whole, exercise training has a therapeutic role in CVD and can significantly attenuate the atherosclerotic process through its beneficial effects on endothelial function and cardiovascular system.

### Conflict of interest statement

The authors declare that the research was conducted in the absence of any commercial or financial relationships that could be construed as a potential conflict of interest.

## Abbreviations

ACS: acute coronary syndrome
AMI: acute myocardial infarction
AR: adrenergic-receptors
BM: bone marrow
CAD: coronary artery disease
CFU-Hill: colony forming unit-Hill
CRP: C-reactive protein
CVD: cardiovascular disease
e-NOS: endothelial-nitric oxide synthase
EPC: endothelial progenitor cell
EST: exercise stress test
GPx-1: glutathione peroxidase type-1
HIF-1: hypoxia-inducible factor-1
HF: heart failure
LVEF: left ventricular ejection fraction
MMP-9: matrix metallopeptidase-9
MNC: mononuclear cell
NO: nitric oxide
PAD: peripheral artery disease
ROS: reactive oxygen species
SDF-1: stromal cell-derived growth factor-1
SOD: superoxide dismutase
VEGF: vascular endothelial growth-factor
TNF-α: tumor necrosis factor- α
